# Acid-sensing ion channels: dual function proteins for chemo-sensing and mechano-sensing

**DOI:** 10.1186/s12929-018-0448-y

**Published:** 2018-05-24

**Authors:** Yuan-Ren Cheng, Bo-Yang Jiang, Chih-Cheng Chen

**Affiliations:** 1 Department of Life Science, National Taiwan University, Taipei, 106 Taiwan; 2 Institute of Biomedical Sciences, Academia Sinica, 128, Academia Rd. Sec. 2, Taipei, 115 Taiwan; 3 Taiwan Mouse Clinic – National Comprehensive Mouse Phenotyping and Drug Testing Center, Academia Sinica, Taipei, 115 Taiwan

**Keywords:** ASIC, ASIC3, Mechanotransduction, Nociceptor, Pain, Proprioception

## Abstract

**Background:**

Acid-sensing ion channels (ASICs) are a group of amiloride-sensitive ligand-gated ion channels belonging to the family of degenerin/epithelial sodium channels. ASICs are predominantly expressed in both the peripheral and central nervous system and have been characterized as potent proton sensors to detect extracellular acidification in the periphery and brain.

**Main body:**

Here we review the recent studies focusing on the physiological roles of ASICs in the nervous system. As the major acid-sensing membrane proteins in the nervous system, ASICs detect tissue acidosis occurring at tissue injury, inflammation, ischemia, stroke, and tumors as well as fatiguing muscle to activate pain-sensing nerves in the periphery and transmit pain signals to the brain. Arachidonic acid and lysophosphocholine have been identified as endogenous non-proton ligands activating ASICs in a neutral pH environment. On the other hand, ASICs are found involved in the tether model mechanotransduction, in which the extracellular matrix and cytoplasmic cytoskeletons act like a gating-spring to tether the mechanically activated ion channels and thus transmit the stimulus force to the channels. Accordingly, accumulating evidence has shown ASICs play important roles in mechanotransduction of proprioceptors, mechanoreceptors and nociceptors to monitor the homoeostatic status of muscle contraction, blood volume, and blood pressure as well as pain stimuli.

**Conclusion:**

Together, ASICs are dual-function proteins for both chemosensation and mechanosensation involved in monitoring physiological homoeostasis and pathological signals.

## Background

Sensing tissue acidosis and mechanical force is essential for an organism to respond to noxious stimuli and/or physiological changes for survival [1, 2]. In vertebrates, primary sensory afferents of dorsal root ganglia (DRG), trigeminal ganglia (TG), and nodose ganglia (NG) project to tissues all over the body to detect tissue acidosis and monitor force changes from outside or inside the body [3, 4]. Tissue acidosis occurs during tissue injury, inflammation, ischemia, and metabolic changes, whereas force changes arise from tactile stimuli, muscle contraction, visceral organ movement, tooth movement, blood and body fluid flow, etc. Chemoreceptors or metaboreceptors are sensory neuron subtypes responsible for sensing tissue acidosis; mechanoreceptors are the other subtypes for sensing force. In addition, polymodal nociceptors detect noxious tissue acidosis and noxious mechanical stimuli [5]. Accumulating evidence has revealed membrane proteins that allow sensory neurons to monitor tissue acidosis, including acid-sensing ion channels (ASICs), transient receptor potential (TRP) channels, ATP-gated ion channel (P2X), two-pore domain potassium (K2P) channels, and proton-sensing G-protein–coupled receptors (GPCR; e.g., G2A, GPR4, OGR1, TDAG8) [2, 6, 7].

However, mechanically activated ion channels that allow sensory neurons to transmit mechanical stimuli are not fully understood. Candidate mechanically activated ion channels include ASICs, K2P channels, PIEZO proteins, tentonin 3, transmembrane channel-like (TMC) proteins, TRP channels, and volume-regulated anion channel (VRAC) [1, 4, 8, 9]. Some ion channels (e.g., ASICs, K2P, and TRP channels) seem to have dual protein functions in sensing both tissue acidosis and mechanical force. In this review, we focus on recent progress in research into ASICs and discuss how these ion channels are involved in acid-sensing and mechano-sensing functions of sensory neurons.

### Acid-sensing ion channels

**Fig. 1:**
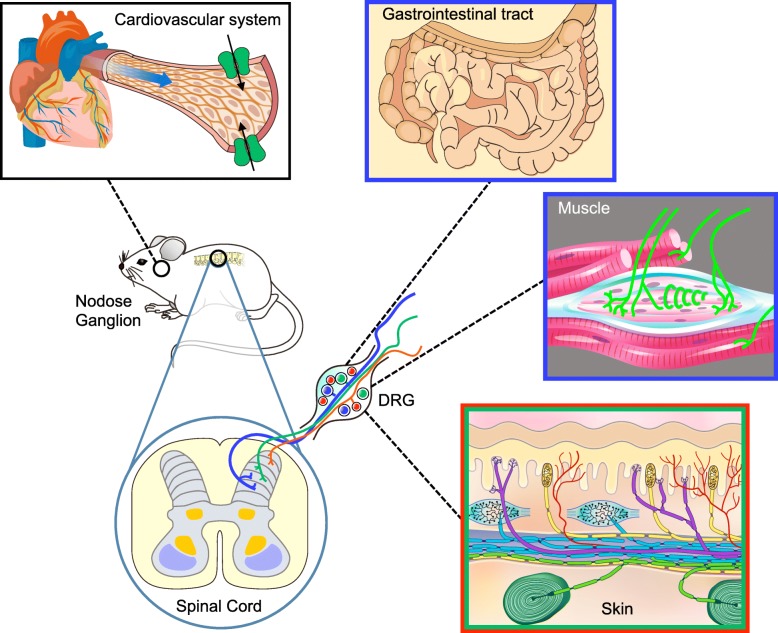
ASICs localize in a variety of somatosensory nerve terminals responsible for both chemo-sensing and mechano-sensing functions. In skin, ASICs are expressed in the free nerve endings of nociceptors and cutaneous nerves projecting to specialized mechanoreceptors such as Meissner corpuscles, Ruffini corpuscles, Pacinian corpuscles, hair follicles, and Merkel cells. In muscle, ASICs are expressed in the free nerve endings of nociceptors and group Ia muscle spindle nerve fibers in the intrafusal bag. In the cardiovascular system, ASICs are expressed in cardiac sensory nerves and baroreceptors. In the gut, ASICs are expressed in many subpopulations of gastrointestinal afferents. In the spinal cord, ASIC-expressing sensory afferents innervate to distinct dorsal horn laminae corresponding to their specific sensory perception

ASICs are members of the degenerin/epithelial sodium ion channel (DEG/ENaC) family, a group of voltage-insensitive cation channels expressing in the nervous system and many types of epithelial cells and immune cells [10] (Fig. 1). In rodents and humans, four genes, including *Accn1*, *Accn2*, *Accn3*, and *Accn4*, encoding at least six ASIC subtypes (ASIC1a, ASIC1b, ASIC2a, ASIC2b, ASIC3, and ASIC4) have been reported [11, 12]. ASICs are known as proton-gated ion channels, because heterologously expressed ASIC1a, ASIC1b, ASIC2a, and ASIC3 are mainly permeable to Na^+^ (and to a lesser extent Ca^2+^, K^+^) and mediate a transient or a biphasic (transient and sustained) inward current in response to external acidification.

Each ASIC subtype contains two transmembrane domains with intracellular N- and C-termini and a large extracellular loop. Crystallization of the chicken ASIC1a channel revealed that three subunits are required to form a functional channel. The subunit composition of a functional channel could be an assembly of three identical ASIC subtypes (homomeric) or a combination of different ASIC subtypes (heteromeric) [13, 14]. Different combinations of ASIC subtypes show different electrophysiology properties. However, the exact ASIC combinations (and ASIC splice variants) in native neurons remain unclear and need further investigation. According to the crystal structure, a hand holding a ball model has been proposed to represent the stereo domain of each subunit [13]. The hand is further divided into an extracellular domain and two transmembrane helixes. A beta-ball surrounded by the finger, thumb, knuckle, and palm, called the acidic pocket, protrudes into the extracellular area to sense the proton and the external stimuli. Two transmembrane helixes insert into a lipid bilayer as the location of the forearm to form the ion channel pore with other subunits.

The expression patterns of each ASIC subtype have been intensively studied with genetic or immunohistochemistry tools in past 2 decades [15] (Table 1). ASIC1a is abundant in the central nervous system (CNS) [16], including most of the brain regions and the spinal cord as well as the peripheral nervous system (PNS) in TG, DRG, NG, and spiral ganglia (SG) [17–19]. With the wide expression profile, ASIC1a has been found involved in modulating neural activity in the brain and detecting noxious acidosis [20]. Besides the nervous system, ASIC1a is also expressed in peripheral tissues including arteries, bone marrow, intestine, tongue, and bladder [21–25]. ASIC1b is specifically expressed in the PNS and involved in pain sensation, but the exact neuron subtypes that express ASIC1b are not well characterized [26, 27]. ASIC2 (including 2 splice variants, ASIC2a and ASIC2b) is expressed in several brain regions, including the amygdala, hippocampus, cerebellum, olfactory bulb, cortex, brain stem and spinal cord [28, 29] as well as in the PNS in DRG [30] and SG [31]. ASIC2 is largely expressed in mechanoreceptors of the somatosensory system [32], such as lanceolate fibers of hair follicles, the Meissner corpuscles, and the Pacinian corpuscles [33] as well as smooth muscles of the aortic arch [34], cerebral artery, and bladder. Thus, much effort has been done to show the involvement of ASIC2 in neurosensory mechanotransduction [30, 34]. As well, ASIC2 is involved in the pressure-induced smooth muscle contraction [35]. ASIC3 is predominantly expressed in DRG (previously called DRASIC), TG, NG, and SG [3, 36]. In the CNS, ASIC3 is expressed only in the mesencephalic trigeminal nucleus (Me5) neurons in the brain-stem region, which are proprioceptive neurons [37]. Since neurons in DRG are heterogeneous, further molecular identity studies have revealed that ASIC3 is broadly expressed in both peptidergic neurons (expressing CGRP, substance P) [38], non-peptidergic neurons (bound to isolectin B4), pruitoceptors (expressing MRGPRA3), c-mechanoreceptors (expressing tyrosine hydroxylase), myelinated mechanoreceptors (expressing NF-H, TrkC, or NT-3), and proprioceptors (expressing parvalbumin) [3, 15, 39]. Immunohistochemistry studies revealed ASIC3 expression in free nerve endings of the skin and many types of cutaneous nerves projecting to mechanical sensory structures, including lanceolate fibers, Meissner corpuscles, and Merkel cells [38]. However, retrograde tracing studies combined with whole-cell patch clamp recording demonstrate that functional ASIC3 channels are mostly expressed in muscle afferent neurons of DRG nociceptors and proprioceptors [39, 40]. Besides the nervous system, ASIC3 is also expressed in specific peripheral tissues and cells such as bone marrow [41], intestine, adipose tissues, bladder [22], and joint [42]. However, the functional implication of ASIC3 in these tissues requires further investigation. ASIC4 is mainly expressed in the brain and pituitary gland and in prenatal or neonatal DRG [43, 44]. In the brain, ASIC1a, ASIC2 and ASIC4 are all found in oligodendrocyte-lineage cells [15, 45].

**Table 1:** Expression of ASICs in specific subsets of mechanoreceptors

	ASIC1	ASIC1a	ASIC1b	ASIC2	ASIC2a	ASIC2b	ASIC3	ASIC4
Jugular nociceptor	SC-RT [125]	SC-RT [125]	SC-RT [125]	SC-RT [125]		SC-RT [125]	SC-RT [125]	
Nodose nociceptor	SC-RT [125]			SC-RT [125]	SC-RT [125]		SC-RT [125]	SC-RT [125]
Esophageal mechanoreceptor	SC-RT [125]	SC-RT [125]	SC-RT [125]	SC-RT [125]	SC-RT [125]	SC-RT [125]	SC-RT [125]	SC-RT [125]
Muscle spindle			SC-RT [40]	IHC [126]		SC-RT [40]	IHC [40] Reporter [40] SC-RT [40]	
Hair follicle: Lanceolate complex				IHC [30], IHC [32]			IHC [38]	
Hairy skin: Penicillate nerve				IHC [32]				
Meissner’s corpscles				IHC [32]			IHC [38]	
Merkel cell/neurite complex				IHC [32]			IHC [38]	
Cutaneous nociceptor				IHC [32]			IHC [38]	
Baroreceptor	IHC [34]			IHC [34]			IHC [34] IHC [72]	
Periodontal ligament Ruffini’s ending							IHC [36]	

*IHC* immunohistochemistry, *SC-RT* single cell RT-PCR

### Physiological and behavioral phenotyping of ASIC-knockout mice

The genetic approach has been extensively used to probe the physiological and behavioral phenotypes in mice lacking specific ASIC subtypes and has shed light on the roles of ASICs in chemo-sensing and mechano-sensing functions in the nervous system (Table 2), and the causal role of ASIC deficits in the etiology of neurological disorders (Table 3) [15]. Neuron injury is attenuated in *Asic1a*-knockout (*Asic1a*^*−/−*^) mice after ischemic stroke [46], traumatic brain injury [47], and experimental autoimmune encephalitis [48], which suggests a role for ASIC1a in tissue acidosis-induced neuronal excitotoxicity in the brain. Mechanistically, the ASIC1a-mediated calcium conductivity is responsible for neuronal excitotoxicity, which can be inhibited by a selective ASIC1a antagonist PcTx1 [20]. Also, protons are considered potential neurotransmitters activating ASIC1a and thus modulating neural activity and synaptic plasticity in the CNS [49]. Accordingly, *Asic1a*^*−/−*^ mice show impaired neural plasticity in fear circuitry (e.g., amygdala) and reduced fear responses and memory [50]. Of note, amygdala neurons were further found as chemosensors detecting carbon dioxide and acidosis to trigger a fear response [51]. Other related brain behavioral phenotypes of *Asic1a*^*−/−*^ mice include enhanced cocaine-craving behaviors, elevated amphetamine-induced hyperlocomotion, reduced anxiety-like behaviors [15, 52], and increased chemoconvulsant-induced seizure [15, 53]. ASIC1a has roles in the PNS in chemo-sensing and mechano-sensing. In ex vivo studies, *Asic1a*^*−/−*^ mice show enhanced activity of mechanoreceptors in visceral afferent nerves [17]; in pain behavioral studies, *Asic1a*^*−/−*^ mice respond to muscle inflammation and develop secondary (or referred) mechanical hyperalgesia in distal tissues but fail to develop primary mechanical hyperalgesia in muscle [54]. Data for *Asic1b*^*−/−*^ mice are not yet available.

**Table 2:** Effects of ASICs knockout on neural activity of mechanoreceptors

	ASIC1 KO	ASIC2 KO	ASIC3 KO	ASIC2/3	ASIC1/2/3
Nodose arterial baroreceptor		⇎ [34]			
Gastroesophageal mechanoreceptor	Tension receptor ↑ [17, 127] Mucosal receptor ↑ [30]	Tension receptor ↓ [30] Mucosal receptor ↑ [30]	Tension receptor ↓ [30]		
Colonic mechanoreceptor	Mesentric afferent ↑ [30] Serosal afferent ↑ [30]	Mesentric afferent⇋ [30] Serosal afferent ↑ [30]	Mesentric afferent ↓ [30] Serosal afferent ↓ [30] Colonic nociceptor ↓ [70]		
Cochlear mechanoreceptor		⇋ [55]			
Muscle spindle mechanoreceptor			⇎ [40]		
Cutaneous RA mechanoreceptor		↓ [30] ⇋ [55]	↑ [38]		⇋ [77]
Cutaneous SA mechanoreceptor		↓ [30]	⇋ [38]		⇋ [77]
Cutaneous A-fiber mechanonociceptor			↓ [38]		↑ [77]
Down-hair mechanoreceptor			⇋ [38]		⇋ [77]
Cutaneous afferent C-nociceptor			⇋ [38]		⇋ [77]
DRG-RA MA-current neurons		⇋ [114]	⇋ [114]	⇋ [114]	
DRG-IA MA-current neurons		⇋ [114]	⇋ [114]	⇋ [114]	
DRG-SA MA current neurons		⇋ [114]	⇋ [114]	⇋ [114]	
DRG- Proprioceptor			Tether model ↓ [40] Bilayer model ⇋ [40]		

*IA* intermediately adapting, *RA* rapidly adapting, *SA* slowly adapting, *MA* mechanically activated

⇋, no change; ↑, increased activity; ↓, decreased activity; ⇎, impaired or disturbed mechanotransduction

**Table 3:** Effects of ASIC-subtype knockout on neurological disorders

	ASIC1a KO	ASIC2 KO	ASIC3 KO	ASIC4 KO	References
Ischemic stroke	Reduced ischemic brain injury [46]; Reduced acidosis-induced neuronal cell death [131]	NA	NA	NA	[46, 131]
Learning and memory	Impaired fear memory [44]; Impaired spatial memory [133]; No effect on spatial memory [134]	NA	No effect [37]	No effect [44]	[37, 44, 133, 134]
Multiple sclerosis	Reduced EAE-induced clinical symptoms [48, 129, 130]	No effects [129]	No effect [129]	NA	[48, 129, 130]
Epilepsy	Impaired seizure termination and increase kanic acid-induced seizure [44, 53]	NA	NA	No effect [44]	[44, 53]
Anxiety / Fear / Depression /Aggression	Reduced fear and anxiety-like behaviors [51]	NA	Reduced anxiety-like and aggression-like behaviors; no effect on depression-like behaviors [37]	Increased fear and anxiety-like behaviors [44]	[37, 44, 51]
Addition	Increased condition place preference to cocaine and morphine [52]; Increase amphetamine-induced hyperlocomotion, behavioral sensitization, and rewarding [44]	No effect [132]	NA	No effect [44]	[44, 52, 132]
Neuropathic pain	No effect [128, 129]	No effect [129]	No effect [129]	NA	[128, 129]
Fibromyalgia	No effect [61]	NA	No pain developed [61]	NA	[61]
Rheumatic arthritis	Abolished primary hyperalgesia, [42]	NA	Abolished secondary hyperalgesia [42]	NA	[42]
Hearing	NA	Resistant to noise-induced threshold shift [31]	Age-dependent hearing loss to ultrasound [74, 74]	NA	[74, 75]

*NA* not available

In contrast to *Asic1a*^*−/−*^ mice, *Asic2*-knockout (*Asic2*^*−/−*^) mice show major deficits in mechano-sensing phenotypes but no obvious chemo-sensing phenotypes [15]. In ex vivo studies, *Asic2*^*−/−*^ mice show decreased or disturbed neurosensory mechanotransduction of 2 types of cutaneous afferents (rapidly adapting and slowly adapting low-threshold mechanoreceptors) [30], serosal colonic afferents, and tension and mucosal types of gastroesophageal afferents [17]. At a system level, *Asic2*^*−/−*^ mice show impaired baroreceptor reflex [34], impaired pressured-induced constriction in middle cerebral arteries [35], and resistance to noise-induced threshold shifts in hearing [31]. However some conflicting results challenge a role for ASIC2 in neurosensory mechanotransduction with the use of another *Asic2*-knockout design [55]. Further studies with subtype-specific *Asic2a* and/or *Asic2b* models might be helpful to solve the phenotype discrepancy.

Being predominantly expressed in the PNS and the most sensitive proton-sensing ion channel, ASIC3 is proposed to be an important transducer for chemo-sensing, especially for sensing pain associated with tissue acidosis [3]. As well, studies of *Asic3*-knockout (*Asic3*^*−/−*^) mouse phenotype have shown the involvement of ASIC3 in neurosensory mechanotransduction [4, 56]. In chemo-sensing, *Asic3*^*−/−*^ mice lost the acid-induced sustained current in DRG neurons that express ASIC-like currents, whereas in wild-type mice, ~ 20% of DRG neurons expressed ASIC3-like currents containing the sustained component [57]. Accordingly, *Asic3*^*−/−*^ mice show largely reduced acid-induced pain behaviors [58, 59] and impaired triggering of acid-induced nociceptor priming [60]. In a mouse model of fibromyalgia, *Asic3*^*−/−*^ mice failed to develop chronic widespread muscle pain induced by repeated intramuscular injections of acid saline [60–62]. Also, *Asic3*^*−/−*^ mice show impaired triggering of pain associated with tissue acidosis under the conditions of surgery, cardiac ischemia, muscle and joint inflammation, and rheumatoid arthritis [42, 54, 63–67]. In mechano-sensing, *Asic3*^*−/−*^ mice show abnormal neurosensory mechanotransduction of cutaneous and visceral afferents in ex vivo studies of electrophysiological recordings on tissue-nerve preparations [4]. In skin, *Asic3*^*−/−*^ mice show increased activity of rapidly adapting mechanoreceptors and decreased activity of A-fiber mechanoreceptors, so the mice might be more sensitive to light touch and less sensitive to a noxious pinch than wild-type mice [38]. However, inconsistent with the ex vivo studies, *Asic3*^*−/−*^ mice show hypersensitive nocifensive behaviors to dynamic mechanical stimuli [68] and noxious mechanical pressure on the tail [69]. In the viscera, *Asic3*^*−/−*^ mice show decreased activity of tension gastroesophageal afferents and decreased serosal and mesenteric colonic afferents, which suggests that ASIC3 might play a role in visceral nociception [17, 70, 71]. At the system level, ASIC3 is involved in neurosensory mechanotransduction of low-threshold baroreceptors, proprioceptors, skin nociceptors, and the cochlear; indeed, *Asic3*^*−/−*^ mice show deficits in blood volume expansion-induced diuresis [72], proprioceptive behaviors while walking on a balance beam [40], pressure-induced vasodilatation in skin [73], and age-dependent hearing loss [74, 75].

The role of ASIC4 in chemo-sensing and mechano-sensing is still questioned, because ASIC4 itself does not form a functional channel [43]. ASIC4 can downregulate the surface expression of ASIC1a and ASIC3 and thus reduce the acid-induced current of ASICs [76]. Consistently, *Asic4*-knockout (*Asic4*^*−/−*^) mice have shown contrasting behavioral phenotypes to ASIC1a in modulating innate fear and anxiety [44].

These results indicate that ASICs play an important role in chemo-sensing and mechano-sensing in the somatosensory system. Intriguingly, sometimes an ASIC subtype knockout does not downregulate mechano-sensing phenotypes but rather results in enhanced neural activity of cutaneous and/or visceral mechanoreceptors as well as hypersensitive pain responses to noxious stimuli [4]. Also, inconsistent results are found between in vitro, ex vivo, and in vivo assays. For instance, in *Asic3*^*−/−*^ mice, the hypersensitive nocifensive behaviors to noxious mechanical stimuli do not match the reduced activity of A-fiber mechanoreceptors in ex vivo study. Moreover, although A-fiber mechanoreceptor activity is reduced in *Asic3*^*−/−*^ mice and not altered in *Asic1a*^*−/−*^ or *Asic2*^*−/−*^ mice, the *Asic1a*^*−/−*^*::Asic2*^*−/−*^*::Asic3*^*−/−*^ triple knockout results in increased activity of A-fiber mechanoreceptors [77]. Gene redundancy is usually used to explain the unexpected results found in a single gene knockout study, if the knockout gene belongs to a gene family containing other functional homologs. However, the ASIC triple knockout results almost deny the possible gene redundancy, unless there are more functionally active proteins inside the ASIC channel complex playing roles. Alternatively, there might be technical limitations in current approaches to probe the neurosensory mechanotransduction in vitro, ex vivo, or in vivo. One important issue is to know the proper channel-gating model we are investigating when the assays are designed to probe the neurosensory mechanotransduction.

### Molecular basis of chemo-sensing in ASICs

**Fig. 2:**
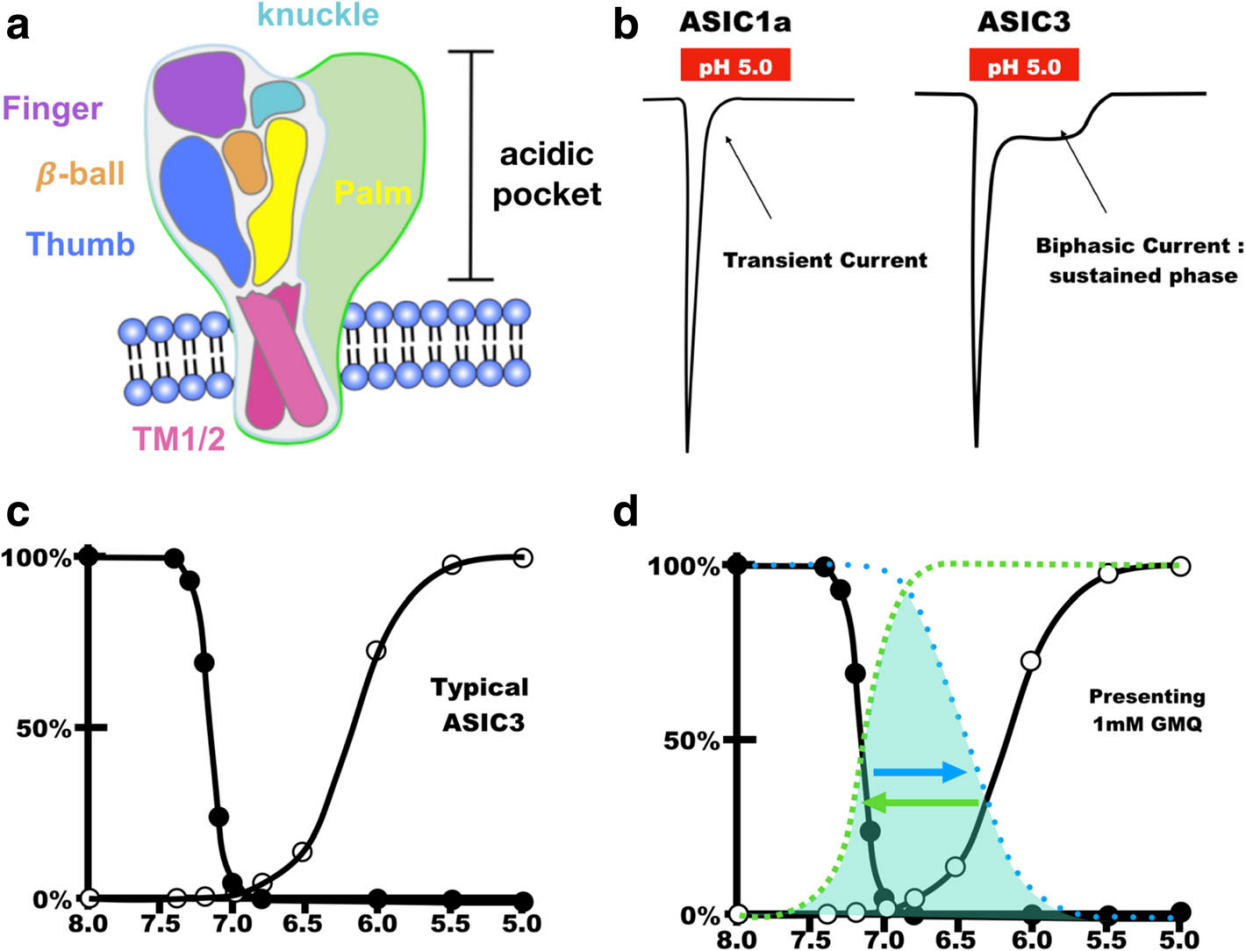
The structure and function of ASICs. **a** A trimeric ASIC channel comprises three subunits. Each subunit constructs “a hand holding a ball” structure to sense the extracellular proton and regulate the proton-gated currents. There are three major categories of non-proton regulators (synthetic/nature compound, endogenous metabolites, and divalent cations) to activate or modulate ASICs in a conformational change of structure. **b** The ASIC1a (or ASIC1b, ASIC2a) homomeric channel mediates a transient current, whereas the ASIC3 homomeric channel mediates a biphasic current containing a transient component followed by a non-inactivated sustained component. **c, d** The non-proton regulators (e.g., 1 mM GMQ) may shift the activation (open circle) or inactivation (filled circle) curve by changing the conformation of ASIC channels and enhance the window current (the shadow region underneath the dotted lines)

Regarding chemo-sensing, ASICs can respond to an external acidic-environment decrease from pH 7.4 to a modest level (pH 6.8) or even harsh acidosis (pH 4.0) [20]. The pH sensitivity of ASIC subtypes is in the order of ASIC3 ≥ ASIC1a > ASIC1b > ASIC2a. Neither ASIC2b nor ASIC4 respond to acidification, but they form functional channels with other ASIC subtypes and thus modulate their pH sensitivity and channel properties. The activation of ASICs induces an inward current to depolarize neurons [78]. From the ASIC1a crystalize structure, an acidic pocket containing several pairs of acidic amino acids has been proposed to be one of the pH-sensors of ASIC channels located at the interface between two subunits, whereas cations may access the channel from the lateral wrist region, then move into a broad extracellular vestibule and reach the channel pore region [79] (Fig. 2a). When ASICs are activated, the inward current desensitizes rapidly, except for ASIC3, which mediates both a rapidly desensitized current and an un-inactivated sustained current during the acidic challenge period (Fig. 2b). The sustained ASIC3 current results from the window of overlap between inactivation and activation of the transient component, which means the sustained current is a result of steady state desensitization. The sustained component of ASIC3 is important for the perception of primary inflammatory pain [58, 80]. The regulation of steady-state desensitization could be further analyzed by fitting the Polymodal Monod-Wyman-Changeux Model with its dose–response curve, by which the slope (Hill coefficient) of the dose–response curve for one stimulus can be pursued to define the intrinsic property of a channel [81]. While the transmembrane domain 1 of ASIC3 and ASIC1a is swapped, the ASIC1a/ASIC3 chimera did not change the Hill coefficient or the pH_50_ of the activation of ASIC1a but strongly promoted the sustained current by stabilizing the open state of channels [82]. Further studies of ASIC1a have revealed that the extracellular linker regions and acidic residues in the acidic pocket are not essential for channel activation but affect the desensitization kinetics and thus control the sustained current. These mechanistic insights could provide opportunities for fine-tuning the pH dependence of ASICs [83, 84].

Besides the structure–activity relationship, pharmacology studies have largely advanced our understanding of chemo-sensing of ASICs [12]. For instance, amiloride is a pan-ASIC reversible blocker inhibiting only the transient current but not the sustained component. However, paradoxical results have shown that high concentrations of amiloride are able to open the homomeric ASIC3 channels or heteromeric ASIC3/ASIC1b channels at neutral pH and mediate a sustained current, synergistically enhancing the channel activation induced by acidosis [10, 85]. More recently, a small synthetic molecule, 2-guanidine-4-methylquinazoline (GMQ), was found to activate and modulate the ASIC3 channel at neutral pH by affecting the desensitization [86]. Besides the synthetic compounds, ASICs are also sensitive to divalent cations. For instance, the neurotoxic metal ion Pb^2+^ reversibly and dose-dependently inhibits ASIC currents in neurons of the PNS and CNS [87]. ASIC1a, ASIC1b, and ASIC3 subtypes are all sensitive to Pb^2+^ inhibition via the binding of the extracellular loop, which is independent of amiloride/Ca^2+^ blockade.

As mentioned previously, ASICs can be activated by synthetic compounds such as aimloride and GMQ at neutral pH. The non-acid chemo-sensing property of ASICs is further established by the identification of endogenous ligands from inflammatory mediators. Arachidonic acid and lysophosphatidylcholine (16:0), the metabolites of phospholipids, activate ASIC3 at neutral pH [88]. Both arachidonic acid and lysophosphatidylcholine (16:0) can potentiate an ASIC current by shifting the ASIC3 activation state close to physiological pH 7.4 and increase the window current [89]. Recent studies also identified the endogenous isoquinoline alkaloids, the precursors of endogenous morphine biosynthesis, as the non-proton ligands of ASIC3 [90]. Moreover, alkaloid lindoldhamine isolated from *Laurus nobilis* is another non-proton ligand of ASIC3 opening the channel under neutral or even more alkaline (pH > 7.4) conditions [91].

One of the most important chemo-sensing properties of ASICs is probably the sensing of lactate. Anaerobic metabolism occurring during tissue ischemia and/or fatiguing exercise results in an accumulation of lactates (up to 15~ 50 mM) and local or systemic lactic acidosis [92]. In the presence of 15 mM lactate, acid (pH 7.0)-induced ASIC-like currents increase about 70% in cardiac ischemia-sensing DRG neurons [93]. Lactate dose-dependently enhances ASIC1a and ASIC3 activity via chelating extracellular divalent cations such as Ca^2+^ or Mg^2+^. Moreover, lactate specifically enhances the sustained ASIC3 current at pH 7.1~ 7.2 but not pH 7.0 and below [94]. The lactate modulation can work on ASICs in excised membrane patches, which suggests that the process does not involve a separate receptor or signaling cascade. Lactic acid is among several compounds triggering angina chest pain or ischemic muscle pain. In ischemic pain, lactate modulation is an example of heterosensitization, in which a stimulus (lactate) increases nociceptor sensitivity without directly activating the transducer (ASICs on ischemia-sensing neurons). Accordingly, homomeric ASIC3 (in rats) and heteromeric ASIC2/ASIC3 (in mice) are the major chemo-sensing transducers in cardiac ischemia-sensing afferent neurons [95, 96].

Together, the activation and desensitization of ASICs involve a variety of responses with excellence flexibility to present the environmental change without changing the channel itself (Fig. 2c,d).

### Mechano-sensing mechanism of ASICs

Although accumulating evidence from genetic studies has suggested that ASICs are mechanical transducers involved in neurosensory mechanotransduction, the mechanical gating mechanism of ASICs is still unclear [4]. The Nobel laureate Martin Chalfie proposed a MEC-4/MEC-10 model (or “tether model”) to describe the molecular mechanism of the mechano-sensing apparatus and the putative gating mechanism in *Caenorhabditis elegans* touch receptor neurons (TRNs) [97]. Genetic screening of mechanosensory abnormality (mec) mutants in the nematode revealed a series of mec genes that encode proteins involved in the mechano-sensing apparatus of TRNs. The TRN mec-encoding proteins include mechanically activated ion channels, extracellular matrix proteins, cytoskeleton, and accessory proteins. Accordingly, the mechanically activated ion channel MEC-4 was first discovered in 1981, and later MEC-10 was found, both of which are the nematode homologs of ASICs [98], belonging to the DEG/ENaC family [99, 100]. To transduce the mechanosensation of TRNs, MEC-4 and MEC-10 proteins need to collaborate with other force-transmitting molecules such as extracellular matrix, cytoskeleton, and accessory proteins. The extracellular matrix proteins include MEC-1 and MEC-9 (proteins containing multiple kunitz and epidermal growth factor-like domains), and MEC-5 (collagen-like protein), which are proposed to receive mechanical stimulus and transfer the force to the tethered, mechanically activated ion channels. The cytoskeleton proteins MEC-7 (α-tubulin) and MEC-12 (β-tubulin) can form protofilament microtubules that are important for force transmission and they maintain specialized structures of mechanosensory puncta. These elastic bundles of extracellular matrix and cytoskeleton proteins may directly or indirectly link with the mechanically activated ion channels MEC-4/MEC-10 to propagate the force and activate the channels. Furthermore, many accessory proteins such as MEC-2 (stomatin-like protein containing a prohibitin domain) and MEC-6 (paraoxonase-like protein) are essential for full functionality of touch sensation in nematodes. The central stomatin-like domain of MEC-2 is predominantly palmitoylated and associated with lipid rafts. Notably MEC-2 provides an important structural scaffold for interaction between MEC-4/MEC-10 proteins and the surrounding lipids to work as a gating-spring for mechanotransduction. Together, these accessory MEC proteins involved in the mechano-sensing apparatus can directly or indirectly tether to the mechanically activated ion channels (MEC-4/MEC-10) and thus deliver the mechanical stimulus from the external environment to open the channels. The gating mechanism of the mechano-sensing apparatus is thus named the “tether model”, which emphasizes the extracellular and cytoplasmic tethers acting like a gating-spring to transmit the stimulus force to the channels [97, 98, 101].

As homologs of MEC-4/MEC-10 in mammals, ASICs interact with stomatin-domain proteins, including stomatin, stomatin-like 1 (STOML1), and somatin-like 3 (STOML3) [56, 102, 103]. These stomatin-domain proteins have different inhibitory effects on acid-induced currents of ASIC1a, ASIC2a, and ASIC3. Especially, STOML3 inhibits acid-induced currents of ASIC2a and ASIC3, and in *Stoml3*-knockout mice, ~ 40% of skin mechanoreceptors are insensitive to mechanical stimuli [104]. Accordingly, *Stoml3*-knockout mice fail to develop mechanical allodynia after neuropathic injury. In mammals, PDZ-domain proteins are required for surface expression of ASICs in neurons. For instance, protein interacting with C kinase 1 (PICK1) modulates the surface expression of ASIC1a, ASIC2a, and ASIC3 in mechanosensory DRG neurons [105–108], whereas Lin-7b and CIPP increase the surface expression of ASIC3 and acid-induced ASIC3 [106, 109]. These PDZ-domain proteins may act as a membrane scaffold via lipid binding to enhance the surface clustering of ASICs and regulate the channel activity. Although they are important for ASIC surface expression, their roles in modulating neurosensory mechanotransduction have yet to be proved. Another PDZ-domain protein, whirlin, is expressed in proprioceptors, a type of low-threshold mechanoreceptor. Mice lacking whirlin show impaired neurosensory mechanotransduction of proprioceptors [110], but the interaction between whirlin and ASICs is not known.

In addition to the MEC-4/MEC-10 model, Lin et al. showed that extracellular matrix and cytoskeleton proteins are essential for mechanotransduction of the tether model in cultured mouse DRG neurons [111]. Moreover, the involvement of extracellular matrix proteins in neurosensory mechanotransduction of mammals is due to RGD ligands targeting on integrins (e.g., collagen; laminin-111, − 211, − 511, and − 332; fibronectin) being able to modulate the mechanically activated responses in the mammalian skin-nerve recording or acute dissociated DRG neurons [112, 113]. However, the direct interaction between RGD-containing extracellular matrix proteins and ASICs requires further investigation.

**Fig. 3:**
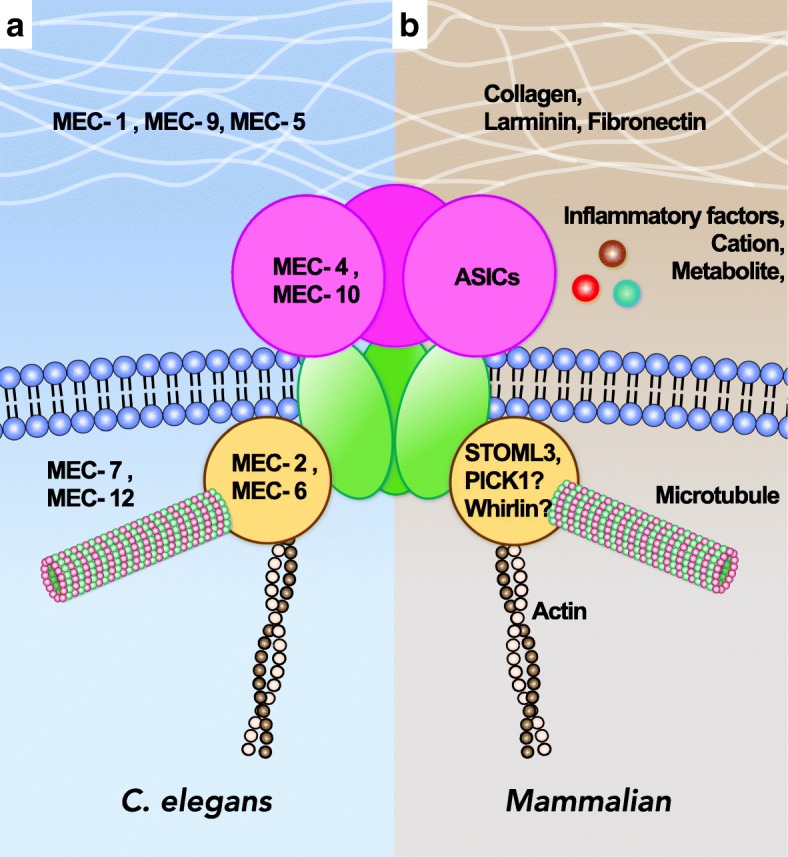
The molecular apparatus that mediates the “tether model” mechanotransduction is conserved between nematodes and mammals. **a** *Caenorhabditis elegans* has many mechanosensory abnormality (*mec)* mutant genes involved in the tether-model mechanotransduction responsible for gentle touch. These *mec* gene products are MEC-4 and MEC-10 (DEG/ENaC channels), MEC-2 and MEC-6 (channel-associated proteins), MEC-7 and MEC-12 (protofilament microtubules), and MEC-1, MEC-5, and MEC-9 (extracellular matrix proteins). These proteins illustrate a tethering gating model of mechanically activated ion channels in nematodes. **b** In mammals, ASICs are homologs of MEC-4 and MEC-10 and are also involved in the tether model of mechanotransduction. ASICs interact with channel-associated proteins PICK1 and STOML3 (and possibly whirlin) and could be regulated by extracellular matrix proteins (e.g., collagen, laminin, and fibronectin) or cytoskeleton proteins such as actin and microtubules

Molecules involved in the tether model of mechanotransduction seem conserved in sensory neurons from nematodes to mammals (Fig. 3). However, although ASICs play an important role in neurosensory mechanotransduction, proving their direct participation in the transduction event like MEC-4/MEC-10 is challenging. In 2004, Drew et al. reported no effects on mechanically activated currents in *Asic2*^*−/−*^, *Asic3*^*−/−*^, and *Asic2*^*−/−*^*::Asic3*^*−/−*^ mice as compared with wild-type littermates in all different DRG neuron populations [114]. Therefore, lack of direct evidence to support ASICs as the mechanically activated channels is a concern in this field. Martin Chalfie raised several challenging issues in discovering a mechanically activated ion channel in eukaryotic organisms, especially in mammals. First, the sensory cells or their receptor endings are sparse, so targeting the mechanical sensors and collecting sufficient number of cells for biochemical assay is difficult. Second, an extremely low amount of the receptor is found. Finally, and perhaps most importantly, assaying the function of candidate transduction molecules in heterologous systems can be difficult. For instance, changing the osmotic pressure of solution can manipulate the membrane tension and is used to identify mechanically activated ion channels (e.g., MscL and MscS of bacteria) that sense forces conveyed through the lipid bilayer [115]. This type of gating mechanism is named the “bilayer model”, which transduces a stimulus modality different from the gating mechanism of the tether model that opens MEC-4/MEC-10 or ASICs via a gating-spring. Another common approach to assay mechanotransduction of the bilayer model in eukaryotic cells (or neurons) is to directly indent the cell surface membrane by using a blunt pipette (mechano-clamp) or pressure jet/suction force to the cell surface (fast-step pressure clamp) [116]. As mentioned previously, Drew and colleagues (in 2004) used the mechano-clamp approach to discount a role for ASICs in this mechanical gating mechanism of the bilayer model. Examining the role of ASICs in mechanotransduction requires assaying the tethered mechanically activated ion channels in vitro, which had not been developed until 2009 [117].

**Fig. 4:**
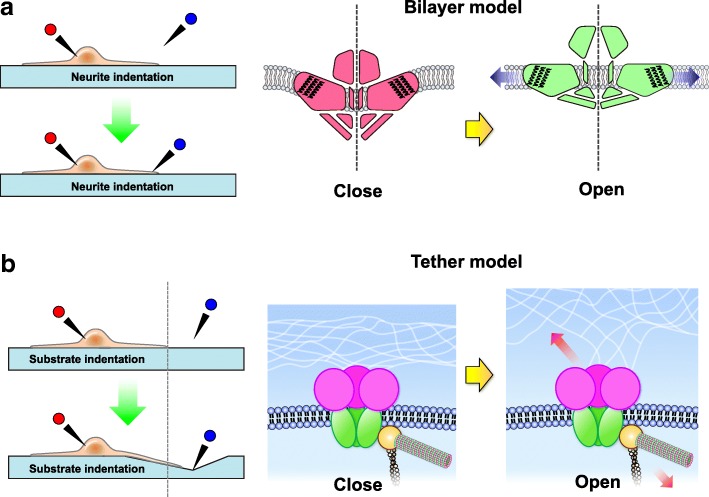
Approaches and mechano-gating mechanisms of the “bilayer model” and “tether model” of mechanically activated ion channels**.** **a** Direct neurite indentation by using a blunt pipette alters membrane tension and thus opens the mechanically activated ion channel of the bilayer model (e.g., PIEZO2). **b** Substrate deformation-driven neurite stretch acts on channel-tethering proteins of the extracellular matrix and cytoskeletons and thus opens the mechanically activated ion channels of the tether model (e.g., MEC-4/MEC-10 or ASICs)

To conquer the limitation of the mammalian nerve system heterogeneity and the challenge of identifying mechanically activated ion channels in eukaryotic organisms, we have successfully developed a novel system to probe localized neural mechanotransduction by using surface-modified elastomeric matrices and electrophysiology, with which we can simultaneously examine the acid-sensing responses and mechanotransduction of the tether and bilayer models in the same cells (Fig. 4) [118]. In this system, we can stretch a single neurite via substrate deformation without contacting the neuron surface to mimic the tethering mechanotransduction of TRN in the nematodes. We thus integrated this elastomeric matrix system and whole-cell patch-clamp recording with ASIC3 conditional knockout and GFP-reporter mice driven by pavalbumin-Cre (representing proprioceptors in the mouse somatosensory system) [40]. We could thus identify ASIC3-expressing DRG proprioceptors via fluorescent microscopy and conduct patch-clamp recording to assay the electrophysiological responses of acid-sensing and mechano-sensing in the same cells. We found that ASIC3 is a functional channel for the acid-induced current in all DRG proprioceptors, although these neurons are known as low-threshold mechanoreceptors. The substrate deformation-driven neurite stretch triggers an action potential or inward current in most DRG proprioceptors, which are largely abolished in mice lacking ASIC3. The result indicates ASIC3 involved in the tether-model mechanotransduction of DRG proprioceptors. However, in *Asic3*^*−/−*^ DRG proprioceptors, direct neurite indentation by using a blunt pipette induces an action potential similar to *Asic3*^*+/+*^ DRG proprioceptors, which suggests that the bilayer model mechanotransduction is still intact. This is the first example with which we can clearly determine a role for ASIC3 directly participating in mechanotransduction in an in vitro assay system. Echoing the MEC-4/MEC-10 model in nematodes, ASIC3-mediated mechanotransduction is specific for the tether but not bilayer model. Moreover, ASIC3 seems to be a dual-function protein in proprioceptors exerting both acid-sensing and mechano-sensing functions. Whether other ASIC subtypes play a role similar to ASIC3 are unknown but remain for further investigation.

### ASICs as tunable somato-sensory transducers

Cells dynamically interact with changes in surroundings by various mechanisms (including chemo-sensing, mechano-sensing, and thermo-sensing) in different timescales. Neurosensory mechanotransduction is extremely fast and too rapid to involve a chemical intermediate, so the electrical response must result from the direct gating of a transduction channel. The discovery of PIEZO proteins as the mechanically activated ion channels of the bilayer model is a milestone in mechanobiology [116]. PIEZO channels fulfill most of the requirements for the true mechanically activated ion channels and were soon found to play important roles in neurosensory mechanotransduction, including proprioception, sensing light touch, and sensing stretch in organs (e.g., lung) [118]. However, PIEZO proteins lack the chemo-sensing function, and their capacity to respond to environmental changes is limited. PIEZO2-mediated mechanically activated currents can be enhanced by STOML3 [119] or GPCR receptor signaling mediated by bradykinin [120], but no chemical mediators have been found to modulate PIEZO protein activity directly. The gating mechanism of PIEZO channels follows the bilayer model, with the mechanical force directly transmitted to the channel via lateral tension in the membrane bilayer. This gating mechanism is firm and rigid and is little tunable by environmental changes.

In contrast, MEC-4/MEC-10 or ASICs are more tunable transducers than PIEZO proteins in response to various somatosensory stimuli. In nematodes, although MEC-4 is the major component of the conductive ion channel, MEC-10 still plays a regulatory role in the mechanical sensing complex [121]. In mammals, ASIC3 is a major component responsible for tether-model mechanotransduction in DRG proprioceptors [40]. ASIC3-containing channels are heterogeneous and could be assembled as an ASIC3 homomeric or heteromeric trimer with ASIC1a, ASIC1b, ASIC2a, or ASIC2b, with distinguishable channel kinetics and modulated by different chemical mediators. Thus, ASICs may work as a tunable unit in parallel to PIEZO2 in neurosensory mechanotransduction in mammals. For instance, PIEZO2 plays a major role in mechanotransduction of low-threshold mechanoreceptors for proprioception and sensing light touch, whereas ASICs show indispensable functionality in regulating mechanotransduction in the same sensory neurons [40, 122–124]. As discussed previously, the channel kinetics of ASICs in chemo-sensing could be altered by many endogenous environmental changes, including acidosis, metabolites, extracellular cations, cytokines and inflammatory factors, and thus reveal ASICs as important transducers of peripheral sensitization in the somatosensory nervous system. Whether these environmental factors could modulate the mechanically gating property of ASICs is intriguing and requires further investigation. In addition, as a channel for tether-model mechanotransduction, mechanical strikes by the extracellular matrix, membrane-associated protein, and cytoskeleton could further fine-tune the already tunable ASICs and provide excellent flexibility for mechanotransduction in mammals.

## Conclusions

In conclusion, we are just beginning to appreciate the tunable function of ASICs and the dual-function roles of ASIC3 in chemo-sensing and mechano-sensing. Still, several basic questions about the roles of ASICs in mechano-sensing functions remain unsolved. First, we are yet to reproduce the ASIC-mediated mechanically activated current in a heterologous expression system. To reproduce the mechanosensitivity of ASICs in non-neuronal cells could be challenging, because the tether-model mechanotransdution can be only assessed in a neurite-like structure and the mechanotransducer might be a complex of the transducer channel and many accessory proteins, plus specific ECM components. Much effort is needed to identify the important molecules involved in the mechanotransducer complex. Second, we are yet to know the roles of each ASIC subtype and the heteromeric ASIC channels in mechano-sensing and related biological functions, such as the modulation of mechanical sensitivity by endogenous metabolites or the other extracellular ligands. Furthermore, the exact combination of ASIC subtypes for an endogenous ASIC channel is not clear. Finally, it is still not known whether the mechano-sensing function of ASICs is involved in neurological disorders beyond proprioception, especially in the central nervous system. For instance, in the context of stroke, both acid-sensing and mechano-sensing functions of ASICs might play roles in response to ischemia and increased intracranial pressure. However, as shown in the Table 3, most neurological disorders seem to be related to the acid-sensing function of ASICs. Future research on each ASIC subtype will continue to pave the way to a better understanding of our somatosensory system and its importance in regulating biological processes in response to external and internal challenges.

## Abbreviations

ASIC3: Acid-sensing ion channel 3
Asic3^−/−^: Acid-sensing ion channel 3 knockout
ASICs: Acid-sensing ion channels
CNS: Central nervous system
DEG/ENaC: Degenerin/epithelium sodium channel
DRG: Dorsal root ganglia
mec: Mechanosensory abnormality
NG: Nodose ganglia
PNS: Peripheral nervous system
SG: Spiral ganglia
STOML1: Stomatin-like 1
STOML3: Stomatin-like 3
TG: Trigeminal ganglia
TRN: Touch receptor neuron
